# Prediction of brain-computer interface aptitude from individual brain structure

**DOI:** 10.3389/fnhum.2013.00105

**Published:** 2013-04-02

**Authors:** S. Halder, B. Varkuti, M. Bogdan, A. Kübler, W. Rosenstiel, R. Sitaram, N. Birbaumer

**Affiliations:** ^1^Department of Psychology I, University of WürzburgWürzburg, Germany; ^2^Institute of Medical Psychology and Behavioral Neurobiology, University of TübingenTübingen, Germany; ^3^Wilhelm-Schickard Institute for Computer Science, University of TübingenTübingen, Germany; ^4^Department of Computer Engineering, University of LeipzigLeipzig, Germany; ^5^Department of Biomedical Engineering, University of FloridaGainesville, FL, USA; ^6^Ospedale San Camillo, Laboratorio di Neuroscience Comportamentale, Istituto di Ricovero e Cura a Carattere ScientificoVenezia, Italy

**Keywords:** BCI, motor imagery, aptitude, DTI, fractional anisotropy

## Abstract

**Objective:** Brain-computer interface (BCI) provide a non-muscular communication channel for patients with impairments of the motor system. A significant number of BCI users is unable to obtain voluntary control of a BCI-system in proper time. This makes methods that can be used to determine the aptitude of a user necessary.

**Methods:** We hypothesized that integrity and connectivity of involved white matter connections may serve as a predictor of individual BCI-performance. Therefore, we analyzed structural data from anatomical scans and DTI of motor imagery BCI-users differentiated into high and low BCI-aptitude groups based on their overall performance.

**Results:** Using a machine learning classification method we identified discriminating structural brain trait features and correlated the best features with a continuous measure of individual BCI-performance. Prediction of the aptitude group of each participant was possible with near perfect accuracy (one error).

**Conclusions:** Tissue volumetric analysis yielded only poor classification results. In contrast, the structural integrity and myelination quality of deep white matter structures such as the Corpus Callosum, Cingulum, and Superior Fronto-Occipital Fascicle were positively correlated with individual BCI-performance.

**Significance:** This confirms that structural brain traits contribute to individual performance in BCI use.

## 1. Introduction

Brain-computer interface (BCI) provide a non-muscular control channel that can be used for communication, device control, or rehabilitation. Most non-invasive BCI rely on control signals that are extracted from components of the electroencephalogram (EEG), first described by Berger (1929), that can be voluntarily modulated. One of the earliest control signals used successfully for communication with severely paralyzed amyotrophic lateral sclerosis (ALS) patients were slow cortical potential (SCP) (Birbaumer et al., 1990, 1999). Later studies were often based on event-related potential (ERP), mainly the P300, [(Farwell and Donchin, 1988; Polich, 2007; Nijboer et al., 2008b, Silvoni et al., 2009; Lulé et al., 2013); see Kleih et al. (2011) for a review]. P300 BCI have the advantage of requiring almost no user training. Steady state-evoked potential (SSEP) share this advantage and have also been successfully used for BCI (Regan, 1977; Middendorf et al., 2000; Allison et al., 2008). It is also possible to control a BCI by performing motor imagery of different body parts such as hands or feet which causes event-related desynchronization (ERD) and event-related synchronization (ERS) of the sensorimotor rhythm (SMR) (also referred to as μ-rhythm) (Pfurtscheller and da Silva, 1999; Neuper et al., 2003; Kübler et al., 2005). Novel approach utilize the principles of semantic classical conditioning to establish a communication channel (Furdea et al., 2012; De Massari et al., 2013; Ruf et al., 2013).

In the context of this study BCI paradigms that rely on user training are of particular interest. This includes BCI based on the modulation of SMR or SCP. Of these we will focus on BCI based on the modulation of the SMR using motor imagery. The SMR is a brain rhythm in the frequency range of 8–15 Hz which is insensitive to visual input (Kuhlman, 1978). Distinct rhythms originating from the somatosensory cortex are modulated by executed movement or movement imagery depending on the body part involved in the task (Hari and Salmelin, 1997; Pfurtscheller et al., 1997). In addition to modulation in the alpha band, modulation can also occur in a second peak in the beta band (16–30 Hz). This peak is located anteriorly to the sources of the alpha component of the SMR (Hari and Salmelin, 1997).

Two approaches to achieving high accuracies with SMR bases BCI have been established. Studies more focused on single sessions with high information transfer rate (ITR) with healthy participants use a large number of EEG sensors to which advanced spatial filtering methods such as common spatial patterns (CSP) are applied (Ramoser et al., 2000; Blankertz et al., 2008). This requires fewer sessions of user training. In studies focused on BCI usage with patients multiple sessions should be possible with a minimum preparatory overhead (such as applying a large number of sensors) which is why user training enabling BCI usage with a low number of sensors is the preferred approach (Neuper et al., 2003, 2009; Kübler et al., 2005; Nijboer et al., 2008a). The time of training is one the reasons for the need of a reliable predictor that estimates the aptitude of a potential user to control such a BCI before training. Additionally, current research on the use of EEG-based BCI suggests that a certain percentage of users would not at all be able to gain sufficient voluntary control of an EEG-BCI within an acceptable timeframe: a phenomenon recently referred to as BCI inefficiency (Kübler et al., 2011). The criterion level of free control is defined to be at 70% selection accuracy Kübler et al. (2011, 2004). In Guger et al. (2009) 50% of participants (*N* = 99) did not achieve accuracies above 70% with an SMR based BCI, in Hammer et al. (2012) 37.5% of participants (*N* = 80) failed to achieve accuracies above 70%. The availability of reliable performance prediction methods would also considerably improve the process of selecting the most promising paradigm for a particular user. Additionally, training programs could be adapted to the aptitude of a particular user at a much earlier stage. Considering that BCI are primarily intended for patients who are diagnosed with severe diseases that not only lead to motor impairment but also to a reduced attention span it would be advantageous to be able to quickly choose a suitable BCI and training strategy that best fits the patients needs (Birbaumer et al., 2008). It was shown that the amplitude of the SMR during rest strongly predicts the performance of a participant in a subsequent SMR-BCI session (Blankertz et al., 2010). A study using functional magnetic resonance imaging (fMRI) has shown that there is a strong difference in supplementary motor area (SMA) activation between high and low aptitude SMR-BCI users when performing motor imagery (Halder et al., 2011). This difference is enhanced when the participants observe motor tasks. For P300- based BCI it could be shown that prediction is possible using data collected from auditory oddball ERP recorded before the experiment but also using features extracted during stimulation (Mak et al., 2012; Halder et al., 2013).

In the light of recent studies on the association of bundle specific white matter integrity and EEG features in healthy subjects (Valdés-Hernández et al., 2010), the link of interhemispheric white matter connectivity with EEG frontal coherence (Teipel et al., 2009) and the relation of interhemispheric transfer time to DTI derived measures of white matter integrity, it is apparent that the conduction properties (e.g., conduction velocity, myelinization, and local fiber density) of the brain are determined by the underlying white matter network and that these properties might in turn significantly influence EEG features on both the trait and state level. Specifically, the dynamics of the resting state alpha rhythm appear to be connected to white matter architecture (Valdés-Hernández et al., 2010). Due to the dependence of the resting state SMR that was shown in Blankertz et al. (2010) and the reported link between EEG features in that frequency range and white matter architecture (Valdés-Hernández et al., 2010), we assumed that there may be a link between white matter architecture and SMR-BCI aptitude.

Based on these findings we hypothesized, that structural characteristics of the user such as head size, white matter integrity, or cortical surface area also influence BCI aptitude. We hypothesized that structural differences in the brains of high and low aptitude users can be identified and that these features not only differ between the groups of high and low aptitude users, but are strongly correlated with individual BCI aptitude. In order to investigate this relationship we conducted a single EEG-BCI session and a structural MRI measurement with 20 healthy participants. We refer to the potential skill of BCI usage of an individual as aptitude. The performance achieved by naive BCI users has been shown to be predictive of subsequent peformance in previous studies (Neumann and Birbaumer, 2003; Kübler et al., 2004). Thus, aptitude is used a synonym for the performance in the first BCI session in the context of this paper.

## 2. Methods

### 2.1. Participants

Twenty healthy participants (7 female, mean age 24.5 years, SD ± 3.7, range 19–36) took part in the study which was approved by the Ethical Review Board of the Medical Faculty, University of Tübingen. Each participant was informed about the purpose of the study and signed informed consent prior to participation. Additionally, each participant signed a form informing him or her about potential risks and exclusion criteria of functional magnetic resonance imaging. Participants were payed 8 €/h. All participants had no prior experience with SMR BCI, had no history of neurological diseases and were German native speakers. Psychological measurements before the experiment showed that both groups had equal levels of intelligence using Raven's standard progressive matrices [mean 66.75, (SD ± 19.29), high aptitude users 71.9 (SD ± 8.31), low aptitude users 61.6 (SD ± 25.64), Wilcoxon rank test *p* = 0.4]. The datasets of four participants could not be used in the offline analysis. The dataset of VPTAB was not complete, the anatomical scan of VPTBJ revealed an incidental finding of a brain abnormality (a large portion of the right hemisphere was missing making normalization impossible), a scanner artifact rendered the data from VPTBS useless (frontal signal extinction possibly caused by radio frequency spike artifacts during acquisition due to a mechanical defect) and finally the data of participant VPTBT had missing slices in the structural scan. A detailed overview of EEG-BCI performance, imagery tasks, and demographic data of all participants can be found in Halder et al. (2011). Thus, *N* = 9 subjects were in the low and *N* = 7 subjects in the high aptitude group.

Participants took part in the MRI session depending on individual willingness and suitability. The MRI session was always conducted after the EEG experiement, on average 13.9 days later.

### 2.2 Procedure

#### 2.2.1. EEG-BCI and neurofeedback

Each of the 20 participants performed a single EEG-BCI session. This included measurements in which the participant had to perform motor execution, observation, and imagery. Three imagery measurements which totalled 75 trials of three classes (left hand, right hand, preferred foot) were used to calibrate the feedback parameters. The calibration trials lasted 8 s, in 4 of which the participant performed the task. After calibration three feedback measurements were performed in which the participant had to use the two classes which showed the highest discriminability in the calibration data to control a cursor. Feedback trials had a length of 9 s, 4 of which with feedback. The additional second was used to indicate which class had to be performed in the current trial. In total 300 feedback trials were performed (150 per class). For a summary of the number of trials per task see Table 1. The accuracy the participants achieved in these 300 trials was used to categorize them into high and low aptitude SMR-BCI users by a median split. About 2.5 h were needed for preparation and collecting the calibration data of the system. In total, 5.5–6.5 h were needed to complete the session. For further information and illustrations of the feedback method see Blankertz et al. (2010).

**Table 1:** **Details of EEG experiments**.

**Motor task**	**Trial duration**	**Trials/class**	**Number of classes**
Execution	8 s	25	3
Observation	10 s	20	3
Imagery calibration	8 s	75	3
Imagery feedback	9 s	150	2

Each participant performed motor execution, observation, and imagery used for calibration of the classifier of the BCI and finally motor imagery with SMR-feedback with the optimal combination of two of the three classes (right hand vs. left hand, right hand vs. foot, or left hand vs. foot).

### 2.3. Data acquisition

#### 2.3.1. EEG recording

Participants were seated in a chair approximately one meter away from a digital computer screen on which cues and feedback were displayed. The EEG recording was performed using four 32-channel BrainAmp direct current (DC) amplifiers manufactured by Brainproducts, Munich, Germany. A 128-channel cap manufactured by Easy Cap, Munich, Germany was used. Of these 119 were used for EEG recording and positioned according to the extended 10–20 system (Sharbrough et al., 1991), referenced to the nasion and grounded to an electrode between Fz and Fpz. The EEG was recorded at 1000 Hz, band-pass filtered between 0.05 and 200 Hz and notch filtered at 50 Hz. Electromyogram (EEG) artifacts were monitored with bipolar electrodes on both forearms and the participants preferred leg. EOG artifacts were recorded with electrodes placed above and below the right eye for vertical EOG (superior and inferior orbital fossa), and for horizontal EOG with electrodes placed at the outer canthi of the eyes. This data was used to exclude artifact contaminated trials.

#### 2.3.2. MRI recording

The MRI experiments were performed in a Siemens Magneton Trio Tim 3T whole body scanner using a standard 12-channel head coil. Each subject participated in one DTI measurement (1.8 × 1.8 × 6.5 mm voxels, 5 mm gap, *TR* = 3 s, *TE* = 93 ms, *FoV* = 1150 × 1150, Flip Angle = 90°, 20 transversal slices, 128 × 128 voxels per slice, 20 diffusion directions, *b*-value = 1000 s/mm^2^) with the FoV comprising the full cerebrum and parts of the rostral cerebellum (how much of the cerebellum was included was dependent on individual overall brain size). Anatomical images were acquired using a high resolution T1 sequence (0.5 × 0.5 × 1 mm voxels, 0.5 mm gap, *TR* = 1.9 s, *TE* = 2.26 ms, flip angle = 9°, 176 sagittal slices, 448 × 512 voxels per slice).

### 2.4. Analysis of DTI data

First, the fractional anisotropy (FA) image from the DTI data was calculated for each individual. Subsequently the normalization parameters to Montreal Neurological Institute (MNI) standard space (using the SPM8 echo-planar imaging (EPI) template) were estimated for the FA-image aligned B0-image of the DTI sequence using SPM Version 8. The normalization parameters were then inverted to warp the standard label image of the ICBM-DTI-81 Atlas into each participants original DTI space, avoiding any interpolation of the original FA values. For each of the 50 ICBM-DTI-81 Atlas regions the median of FA values for all voxels with an FA value above 0.25 was extracted and saved in the participant/regional FA value table.

### 2.5. Analysis of anatomical data

We performed a voxel-based morphometry (VBM) Analysis of the T1 weighted anatomical scans to derive descriptors regarding the relative gray matter volume of each Automated Anatomical Labeling (AAL) region as well as the relative white matter volume of each ICBM-DTI-81 Atlas region. The Voxel-based Morphometry Toolbox (VBM5.1) was used for estimation of the individual modulated and unmodulated segmentation outputs. As the modulated outputs can be corrected for non-linear warping only and therefore make any further correction for different brain size redundant, these images can be used directly for volume estimations. For the unified segmentation approach—repeated segmentation, bias correction, and warping iterations as described in Ashburner and Friston (2005)—used in this study the tissue probability maps provided within the SPM5 template set were used because the subjects were drawn from the appropriate population. We applied the thorough clean-up option of the VBM toolbox and a medium Hidden Markov Random Field model for denoising of the T1 image. A check of sample homogeneity of the modulated images [using the standard deviation (SD) approach within VBM5.1] revealed that the VBM results of the images were all within a tolerable range (not considering the previously excluded participants). In order to smooth the resulting images we applied a three dimensional Gaussian smoothing kernel (FWHM = 3 mm, significantly below the rounded cubic root of the volume of the smallest AAL-region in equal voxel-space). Subsequently the images were re-sliced into the 1 × 1 × 1 mm voxel space of the atlas images containing the respective region labels. For each AAL and ICBM-DTI-81 Atlas region the number of gray matter and white matter voxels within the atlas derived volumes of interest was counted—equalling the regional volume of the respective tissue relative to the entire individual brain. These volume values are strongly correlated across our healthy sample as they all measure brain volumes for identical regions. The total raw volume values [ml] for each participant were also extracted, resulting in absolute volume estimates of the individual amount of gray matter, white matter, and cerebro-spinal fluid (CSF) and the respective gray/white matter ratio.

### 2.6. Categorization into high and low aptitude users

The anatomical data extracted with the methods described in the previous sections was used to predict the performance of the participants. Prediction was performed with four distinct feature sets calculated as described in the previous section: relative gray matter volume, relative white matter volume, FA values and total raw volumes of gray and white matter, CSF and gray/white matter ratio. Shrinkage linear discriminant analysis (LDA) was used to classify the participants into high and low aptitude users Blankertz et al. (2011). Participants were assigned to one of the two groups based on their performance in the EEG-BCI experiment. The classifier performance was validated using a leave-one-participant-out cross validation scheme. At the beginning of each cross validation step features were selected from the current training set based on the significance of the correlation (Pearson's *r*, *p* < 0.1) between feature and BCI performance (all excluding the test set). Then the classifier was trained using these features and was applied to the test set (the participant that was not included in the current training). All features were scaled to [0, 1].

## 3. Results

### 3.1. EEG-BCI online accuracy

The median EEG-BCI performance of the 20 participants was at 82.1%. This value was used to split the participants into high and low aptitude BCI users. After the exclusion of the four participants mentioned in the Methods section this resulted in 7 high and 9 low aptitude users. Further details on the sample can be found in Blankertz et al. (2010); Halder et al. (2011), and Hammer et al. (2012).

### 3.2. Performance prediction results

**Figure 1:**
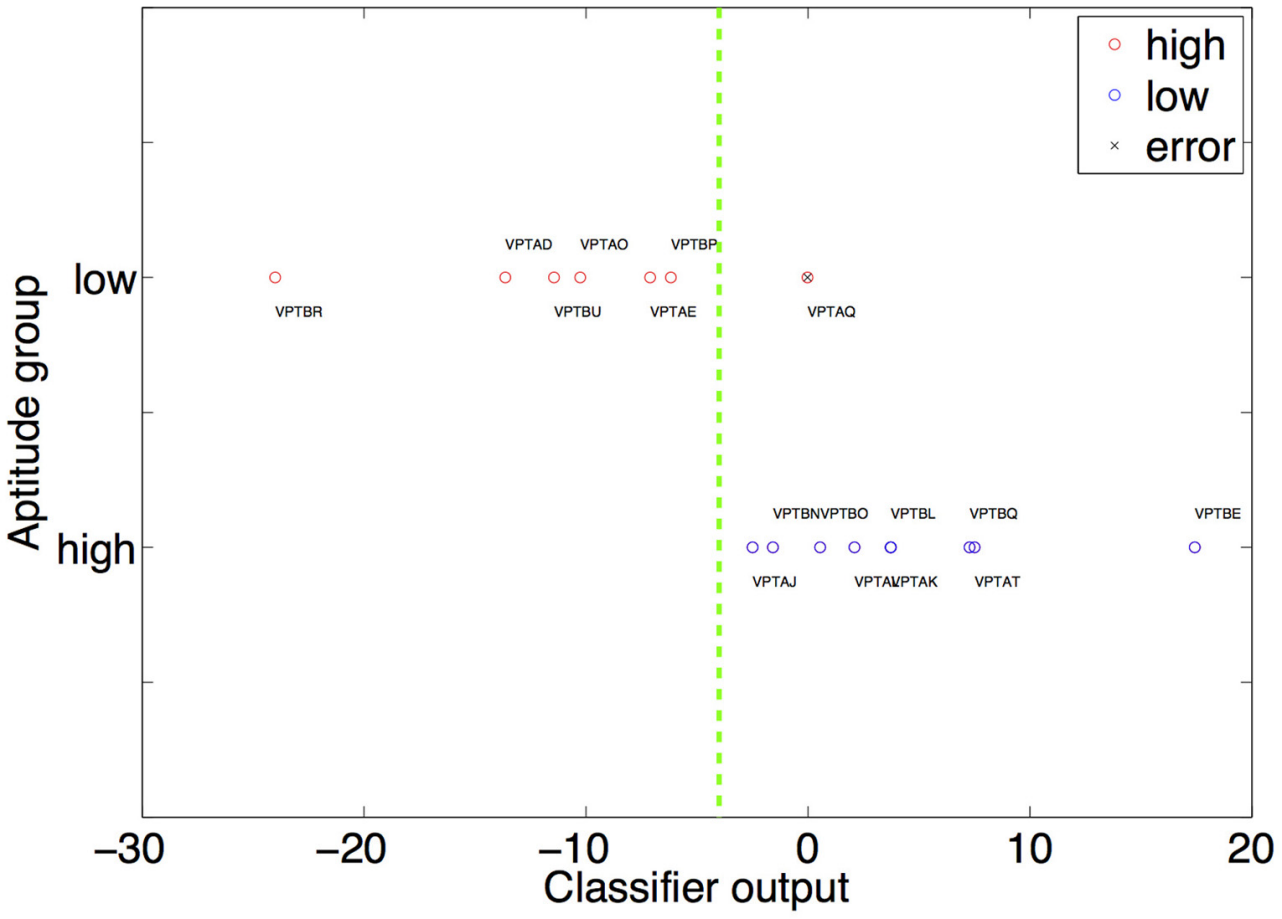
**The result of the multiplication of the weight and feature vector is shown on the x-axis.** The high and low aptitude users are grouped on two separate horizontal lines on the y-axis (red circles high aptitude users, blue circles low aptitude users). This is only used to visually differentiate high from low aptitude users visually. The decision plane used by the classifier therefore has to be a horizontal line (dashed green line). It is optimally placed anywhere between VPTBP and VPTAJ. This placement causes the single error in our classification procedure (prediction of the group of VPTAQ, marked by a black “x”). The weights used to calculate the position of each participant are the ones obtained when this participant comprises the test dataset.

All reported results are based on the leave-one-participant-out cross validation scheme described in the Methods section. If none of the features fulfilled the inclusion criteria in the current step an accuracy of 0.5 (chancel level) is assumed. Using relative gray matter, relative white matter or absolute gray/white matter led only to chance level performance prediction. Using the FA features led to an error of 6.25% (binomial test to quantify significance of prediction *p* < 0.0001). This means that only one participant (VPTAQ, see Figure 1 for a graphical representation of the classifier output) was classified incorrectly. The number of times each feature was selected for classification is given in Table 2.

**Table 2:** **Correlations between FA value and BCI performance of the regions used most often for prediction of the aptitude group**.

**Correlation**	***p*-value**	**Significant**	**ICBM-DTI region code**	**Abbreviation**	**Region**	**Feature usage (%)**
0.63	0.009	Yes	38	CGH-R	Cingulum (Hippocampus) right	100
0.54	0.029	Yes	43	SFO-L	Superior Fronto-Occipital Fasciculus left	100
0.54	0.032	Yes	4	BCC	Body of Corpus Callosum	100
0.52	0.040	Yes	15	CP-L	Cerebral Peduncle left	100
0.51	0.043	Yes	28	PCR-R	Posterior Corona Radiata right	87.5
0.50	0.051	No	34	EC-R	External Capsule right	100
0.48	0.060	No	1	MCP	Middle Cerebellar peduncle	87.5
0.47	0.065	No	16	CP-R	Cerebral Peduncle right	93.75
0.21	0.429	No	17	ALIC-L	Anterior limb of Internal Capsule left	81.25
−0.01	0.956	No	21	RLIC-L	Retrolenticular part of Internal Capsule left	68.75

Only correlations above the second to last horizontal black line are significant (FDR corrected, p < 0.05).

### 3.3. Selected features

**Figure 2:**
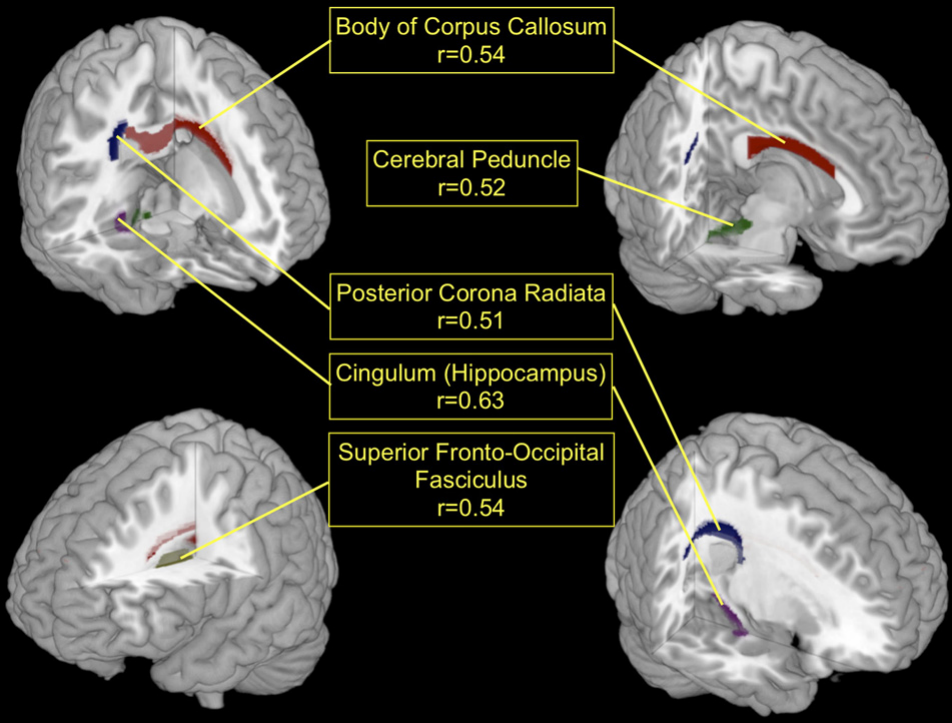
**The top five white matter regions which were most discriminating in the low vs. high BCI-aptitude group comparison (based on feature use over CV-folds) and showed significant correlations (FDR corrected, *p* < 0.05) with individual BCI-performance.** Red, Body of Corpus Callosum; Green, left Cerebral Peduncle; Blue, right Posterior Corona Radiata; Lilac, right Cingulum (Hippocampus area); Yellow, left Superior Fronto-Occipital Fasciculus.

The T1-based anatomical MRI information (rel. GM, rel. WM, and absolute GM/WM/CSF/TIV) could not be used to predict the performance category of the participants of this study. Therefore, all selected features originated from the regionwise extraction of average local FA values for the regions described in the ICBM-DTI-81 Atlas. See Table 2 for the number of times each feature was selected. The areas with highest discrimination were the Body of Corpus Callosum (regioncode 4, selected in 16 CV-folds), the right Cerebral Peduncle (regioncode 15, selected in 16 CV-folds), the right External Capsule (regioncode 34, selected in 16 CV-folds), the right Cingulum at Hippocampus (regioncode 38, selected in 16 CV-folds), the left Superior Fronto-Occipital Fasciculus (regioncode 43, selected in 16 CV-folds), the left Posterior limb of the Internal Capsule (regioncode 16, selected in 15 CV-folds), the Middle Cerebellar Peduncle (regioncode 1, selected in 14 CV-folds), the right Posterior Corona Radiata (regioncode 28, selected in 14 CV-folds), the right Posterior limb of the Internal Capsule (regioncode 17, selected in 13 CV-folds) and the right rentrolenticular part of the Internal Capsule (regioncode 21, selected in 11 CV-folds). Seven further white matter regions (regioncodes 5, 10, 14, 26, 41, 46, and 49) were selected in only one CV-fold. White matter regions with high discriminatory value are illustrated in Figure 2.

### 3.4. Correlation of local fractional anisotropy and EEG-BCI performance

After identifying local FA as the most discriminating feature, the top-discriminating WM regions were selected (based on selection in CV-folds) and local FA values were correlated with individual accuracy (as described in section EEG-BCI and Neurofeedback), as a measure of EEG-BCI performance. The correlations in the Cingulum at Hippocampus, left Superior Fronto-Occipital Fasciculus, Body of Corpus Callosum, left Cerebral Peduncle, and the right Posterior Corona Radiata showed positive correlations (0.51–0.63) with the individual accuracy (FDR corrected, *p* < 0.05). Note that these significances are calculated over the whole dataset whereas the significances used for feature selection were calculated over the subsets of the corresponding cross-validation fold. This can lead to discrepancies such as the FA value of the retrolenticular part of internal capsule left being included in the majority of the folds but still having a large *p-value* if correlated with individual accuracy over full data set. Higher FA in these regions of the brain is related to better individual EEG-BCI performance. This is shown in Figure 2 for the regions whose FA values correlate the strongest with BCI performance. An overview of all regions used during the prediction of the aptitude group is given in Table 2. For comparison purposes we applied the predictor presented in Blankertz et al. (2010) to the data of this study. The resting SMR-based predictor correlates with accuracy with *r* = 0.73 (*p*<0.01) in this sample.

## 4. Discussion

Using only structural MRI data we were able to predict with 93.75% accuracy which aptitude group a participant belonged to according to his or her EEG-BCI performance. This prediction was possible using structural features extracted from DTI images (specifically the FA value), but not using structural features extracted from T1 images. Strong significant correlations between BCI performance and FA values of the region of interest (ROI) defined in the AAL atlas were found for right Cingulum, left superior Fronto-occipital Fasciculus, Body of Corpus Callosum, left Cerebral Peduncle, and right posterior Corona Radiata (see Table 2 for details). Even though the correlation of the FA-values (e.g., the cingulum in Table 2) in this study with BCI accuracy is weaker than the correlation of the SMR amplitude with accuracy (*r* = 0.63 vs. *r* = 0.73) we believe that the presented data is a valuable contribution to the construction of a comprehensive model of BCI performance in addition to the data that is already available (Blankertz et al., 2010; Kleih et al., 2010; Grosse-Wentrup et al. 2011; Halder et al., 2011, 2013; Kaufmann et al., 2011 Hammer et al., 2012). This knowledge can be used to design novel BCI training paradigms which specifically increase the microstructural integrity of central white matter.

Local FA values can be an indicator of myelinization quality, which is critical to the maintenance of appropriate conduction velocities for interregional communication in the brain. It is noteworthy that Valdés-Hernández et al. (2010) report a statistical relation of the spectral position of the alpha peak or the alpha frequency and FA values in a large sample of 222 participants in regions similar to those identified here. Thalamocortical/corticothalamic fibers, commissural fibers, and association fibers such as parts of the Fronto-Occipital Fascicles show a relation to EEG measures also in their sample. In accordance with our findings on TIV and relative volume estimations, purely volumetric anatomical MRI information (head size and neocortical surface area) did not yield any significant relation with the EEG measures in Valdés-Hernández et al. (2010). The probability of an association between EEG phenomena and the structure of thalamocortical connections rather than the thalamus itself, which would have shown in our anatomical volumetric analysis (e.g., through structures such as the thalamocortical parts of the corona radiata) is strengthened by our present data.

Further, both Whitford et al. (2010) and Teipel et al. (2009) report an association of white matter FA in commissural regions with interhemispheric transfer times (Whitford et al., 2010), as well as an association of FA in the middle Cerebellar Peduncle, the Cingulum and frontal and occipital white matter with measures of interhemispheric alpha coherence at various sites (Teipel et al., 2009).

While activity in the alpha-band and the SMR are phenomena which emerge from distinct neurophysiological origins their frequency (Alpha 8–12 Hz, SMR 8–15 Hz) overlaps considerably and both are generated through thalamo-cortical loops. With regard to the strong association of white matter FA and phenomena within these frequencies it is possible, that localized differences in white matter structural integrity are most apparent in these EEG features.

With respect to the SMR, the fact that discriminatory information could be found in the present sample in white matter structures associated with the somatomotoric system (Internal Capsule, Cerebral Peduncle, Middle Cerebellar Peduncle) indicates that microstructural characteristics of the white matter system connecting motor and somatosensory regions within the brain and with the periphery are highly relevant to the formation of an individual SMR and the ability to utilize it for communication and control.

The fact that extra-motoric white matter tracts (e.g., the Fronto-Occipital Fasciculus) also contained information for the discrimination suggests, that these tracts—connecting higher order association cortices—are critical for the large-scale integration and intentional modulation of the SMR via motor imagery. Successful motor imagery requires the recollection of memorized kinesthetic percepts, the assembly of these percepts into a coherent mental image and the ability to intentionally manipulate that mental image by performing sequences of imaginary movements. The microstructure of cingular white matter in the vicinity of the Hippocampus might be responsible for the recollection process, while interhemispheric and fronto-occipital tracts affect imagery and control of that mental image in the frame of the entire BCI task.

Whether such differences—found in the domain of structural traits and functional activation differences (Halder et al., 2011)—all originate from a latent factor which causes these differences and also influences BCI-aptitude, or whether the identified features are of causal relevance for BCI-aptitude themselves can only be resolved by future experiments.

Although based on the present data a link between local FA and BCI performance seems highly intuitive, the underlying causal mechanism remains only poorly understood. In order to investigate a causal link between psychological (Hammer et al., 2012) and neurophysiological predictors of BCI performance with white matter properties we need to record DTI in a larger sample of BCI-users that is as well assessed psychologically. A hypothetical relationship of conduction velocity and local FA could be investigated by combining an interhemispheric transfer time experiment (Whitford et al., 2010) with an assessment of BCI performance. Furthermore, a relationship between EEG features in the Alpha/SMR frequency domain could be characterized further by systematically associating these phenomena with FA measures from a large population, in which the metrics in question vary across a considerable range. More sophisticated DTI measurement schemes with a higher number of applied diffusion directions and better spatial resolution will enable the reconstruction of white matter tracts using tractography methods, which could provide an indication whether the observed differences in local FA merely originate from variations across the dimension of myelinization quality/tissue integrity or whether the anisotropy of some diffusion tensors is reduced due to a higher number of crossing, kissing, or splitting fiber tracts in the voxels of the deep white matter structures in question. Such a finding could indicate more diverse white matter wiring patterns in subjects with low local FA in these structures, rather than indicate variations in myelinization quality. Graph theoretical analysis of white matter connectivity in BCI-users could bring new insights regarding this question and is already beginning to be explored (Buch et al., 2012).

A real time-fMRI training (Caria et al., 2010; Lee et al., 2011) for voluntary up-regulation of SMA-activity (Halder et al., 2011) or extensive training-interventions to increase the FA in the identified white matter regions could yield further insight into the role of these features in the formation of individual BCI-aptitude. Recently it has been shown that extensive training with an EEG-BCI increases motor cortex responsiveness (assessed with transcranial magnetic stimulation) and also the global efficiency index of the scalp electrode connectivity matrix (Pichiorri et al., 2011). It is conceivable that these changes will also be reflected in a change of FA values. Such an association between white matter connectivity features (such as FA) and the factors that influence motor learning (such as SMR features) is subject of ongoing research in the area of stroke rehabilitation (Buch et al., 2012).

One of the central purposes of BCI is their potential to enable communication in patients with progressive degeneration of the motor system such as ALS. While ALS was long considered a disease with mainly motor system specific cerebral involvement, this notion is changing in the light of recent findings on the extensive involvement of extra-motor white matter structures such as the Corpus Callosum (Filippini et al., 2010), Cingulum (Woolley et al., 2011), Uncinate Fasciculus (Sato et al., 2010) or in regions such as the Insula, Hippocampus, the ventrolateral Pre-motor Cortex (PMC), Parietal Cortex, and bilateral Frontal Cortex (Senda et al., 2011). Based on these findings it can be assumed that the pathology in late-stage ALS spreads to multiple central white matter regions, which may be considered critical for the control of those EEG features that are presently used in most BCI applications (e.g., P300, SMR), hence impairing the ability of the patients to utilize present BCI. Birbaumer et al. (2008) proposed that in complete locked-in state (CLIS) output oriented goal directed thinking and imagery impedes and extinguishes instrumental learning of BCI-control leading to an inability of these patients to communicate (Kübler and Birbaumer, 2008). The dysfunctional fiber structure may thus be the consequence or the cause of this deficit.

Fortunately, the notion that white matter microstructure and connectivity no longer change in the adult brain had to be corrected in the light of present findings on the effectiveness of learning interventions such as meditation (Tang et al., 2010), training of working memory (Takeuchi et al., 2010), or juggling training (Scholz et al., 2009) in increasing local FA in certain motor and extra-motor structures—indicating that counter-measures against the deterioration of central white matter in certain pathologies and for the maintenance of individual BCI-aptitude for late-stage communication in ALS should be possible.

Besides for communication, BCIs are being used more and more for other applications such as motor restoration in patients with stroke or other brain damage (Birbaumer et al., 2008; Silvoni et al., 2011). In this field in particular a reliable prediction of BCI aptitude is useful due to the high amount of effort and time involved in this form rehabilitation. In addition to this MRI scans are routinely performed as part of the diagnosis and the DTI data can thus be collected with only a small amount of additional effort. In addition BCI technology has also been used to detect if patients with disorders of consciousness can follow commands, often using MRI, but recently also using EEG (Owen et al., 2006; Lulé et al., 2013). In both usage scenarios, success is already important on a single case basis. Thus, we believe the additional effort of collecting DTI data, that will make successful communication more probable, to be easily justifiable.

The data presented in this paper does not explain 100% of the variance of performance. Thus, other factors besides the ones evaluated here must influence BCI performance. Besides the dependency of performance on brain structure psychological factors such as motivation have been shown to have an influence (Kleih et al., 2010; Hammer et al., 2012). In addition to this physiological traits such as heart-rate-variability or the amplitude of the resting state SMR have been shown to impact BCI performance (Blankertz et al., 2010; Kaufmann et al., 2011). Another aspect of performance will be influenced by more transient factors such as the current level of fatigue or attentiveness. Thus, one limitation of the current study is that the session-to-session stability of the investigated factor was not investigated. Finally, to gain a conclusive predictor of performance all of the aforementioned factors will have to be integrated into a single model.

## 5. Conclusions

Microstructural characteristics of cerebral white matter have a strong (93.75% correct prediction) predictive power of SMR-BCI performance, which may have implications for these training procedures. We can assume that the identified white matter traits will not change within a single session of BCI training. Therefore, our findings indicate that the best strategy of improving BCI performance in low aptitude users is by conducting a long-term BCI training program consisting of multiple sessions, that does not only target to increase proficiency in BCI usage for communication and control but attempts to incorporate interventions that increase or stabilize the microstructural integrity of BCI-critical central white matter.

## Conflict of interest statement

The authors declare that the research was conducted in the absence of any commercial or financial relationships that could be construed as a potential conflict of interest.
